# Supplementary figures and images for: Temporal response patterns of Layer 4 rat barrel cortex neurons across various naturalistic whisker motions
Source: PLoS One. 2024 Dec 23;19(12):e0315887. doi: 10.1371/journal.pone.0315887 (PMC11666059; doi:10.1371/journal.pone.0315887)

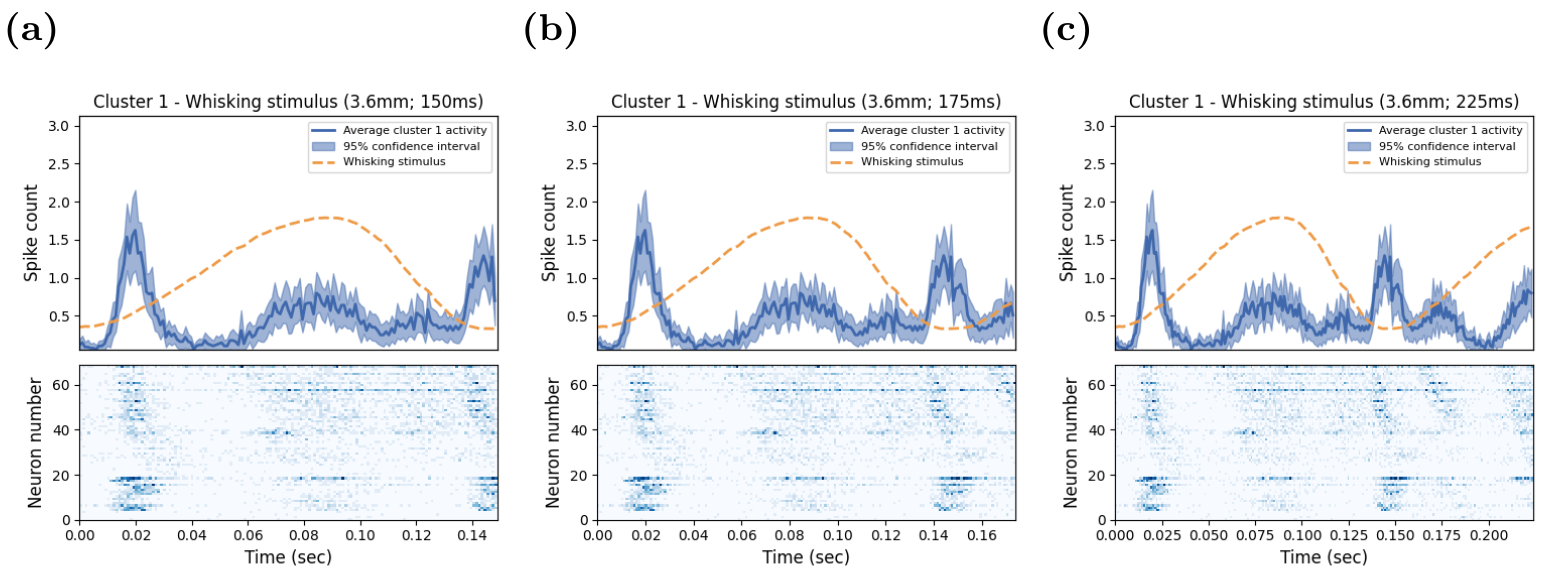

Supplement: S1 Fig — This figure shows the clustering analysis for clustered neural activity in response to the Whisking stimulus at the highest maximum amplitude (3.6 mm) for three different time windows: (a) 150 ms, (b) 175 ms, and (c) 225 ms from stimulus onset. In each panel, the top plot represents the average spike count for neurons in Cluster 1 (solid blue line) along with the 95% confidence interval (shaded blue region), and the Whisking stimulus waveform is overlaid (dashed orange line) for reference. The bottom plot shows the corresponding raster plot of neural activity across the population of neurons in Cluster 1, where each row represents a single neuron. (TIF) [file pone.0315887.s001.tif]

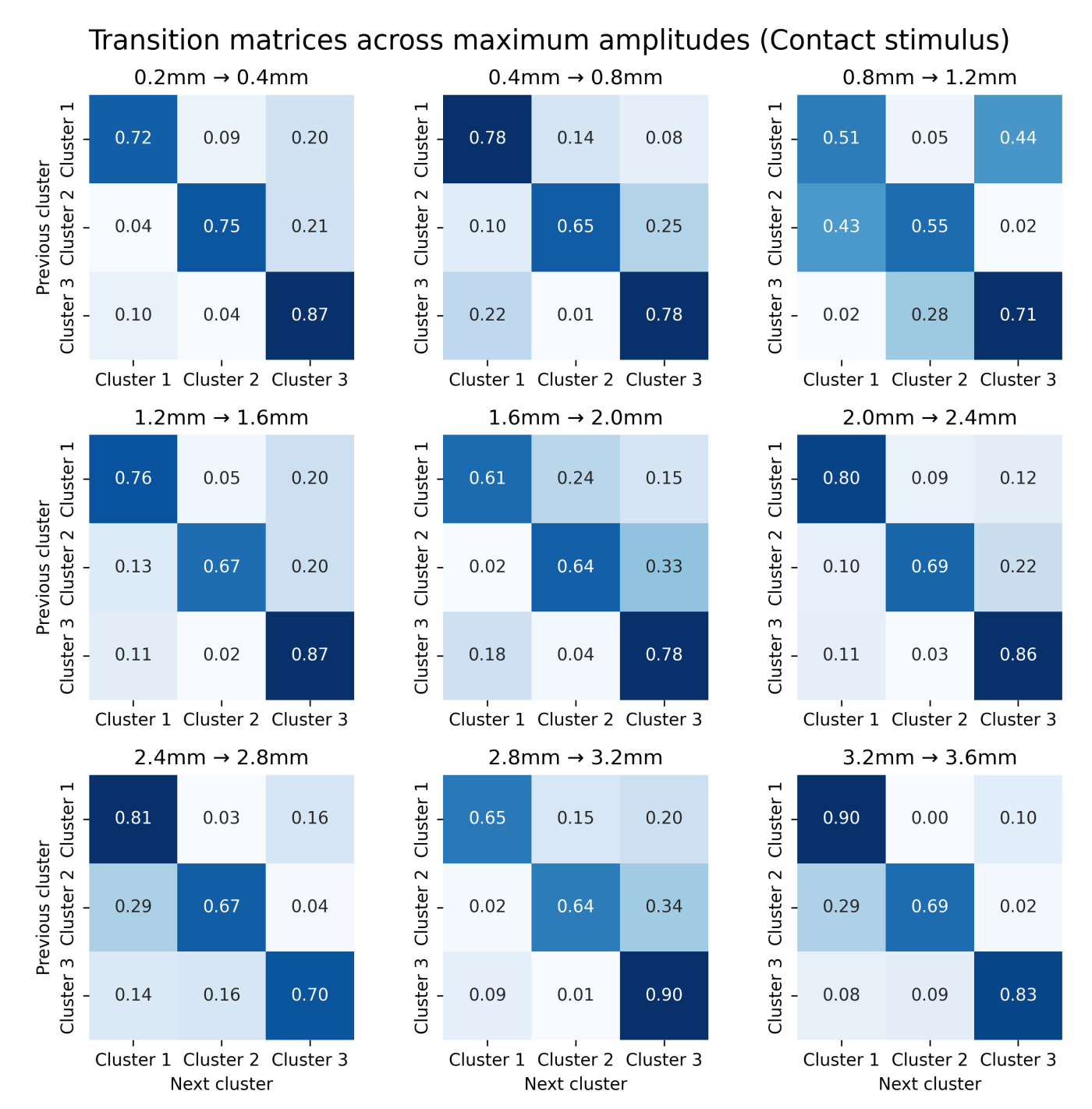

Supplement: S2 Fig — Each heatmap shows the transition probabilities of neurons between clusters across consecutive amplitude levels for the Contact stimulus. The x-axis represents the cluster assignments at the next amplitude, while the y-axis represents the cluster assignments at the previous amplitude. Colours indicate the probability of neurons transitioning between clusters, with darker shades representing higher probabilities. Each transition matrix illustrates how neurons in Cluster 1, Cluster 2, and Cluster 3 change or maintain their cluster membership as the stimulus amplitude increases from one level to the next (0.2 mm → 0.4 mm, 0.4 mm → 0.8 mm, etc.). (TIF) [file pone.0315887.s002.tif]

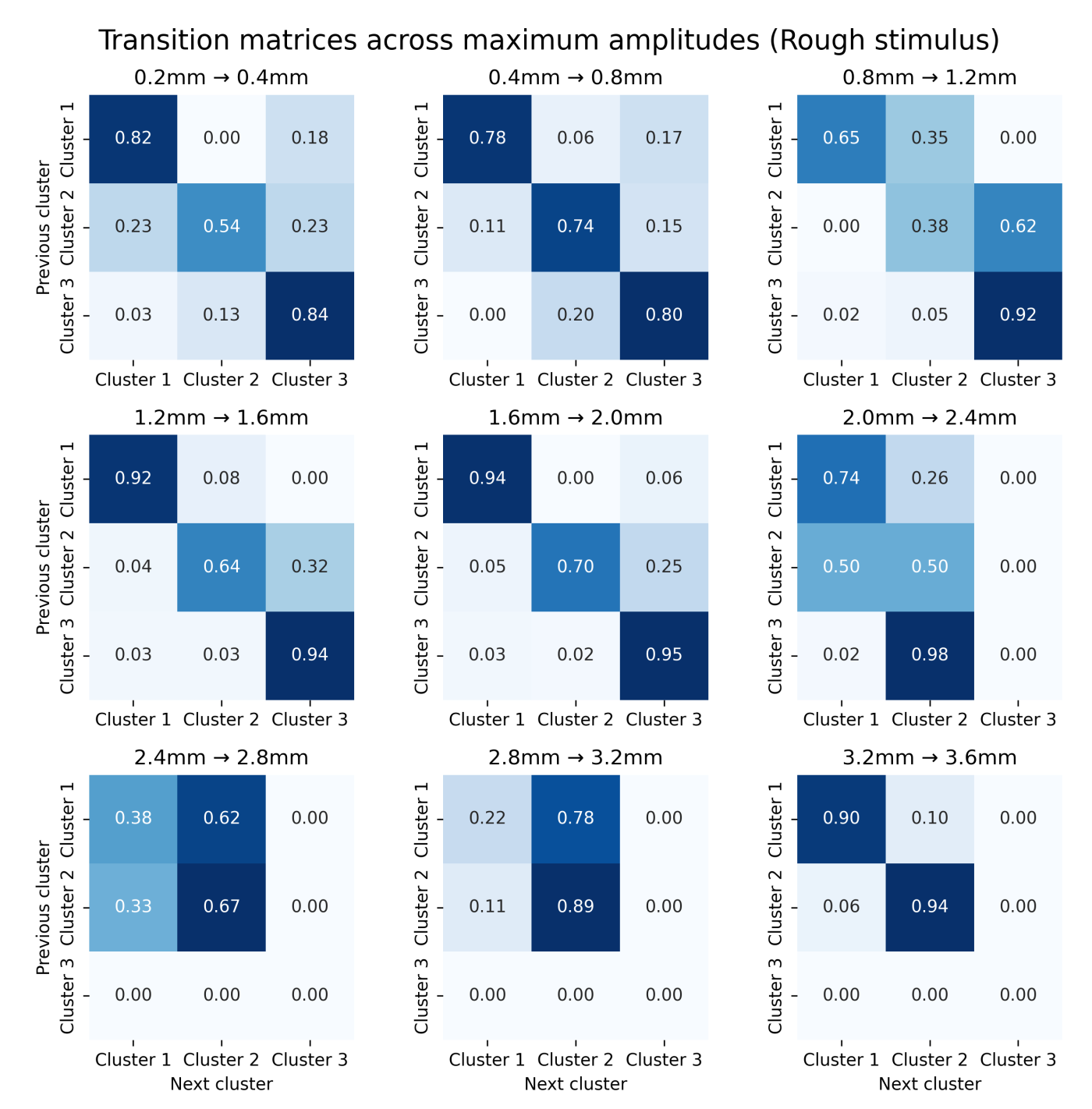

Supplement: S3 Fig — Each heatmap shows the transition probabilities of neurons between clusters across consecutive amplitude levels for the Rough stimulus. The x-axis represents the cluster assignments at the next amplitude, while the y-axis represents the cluster assignments at the previous amplitude. Colours indicate the probability of neurons transitioning between clusters, with darker shades representing higher probabilities. Each transition matrix illustrates how neurons in Cluster 1, Cluster 2, and Cluster 3 change or maintain their cluster membership as the stimulus amplitude increases from one level to the next (0.2 mm → 0.4 mm, 0.4 mm → 0.8 mm, etc.). (TIF) [file pone.0315887.s003.tif]

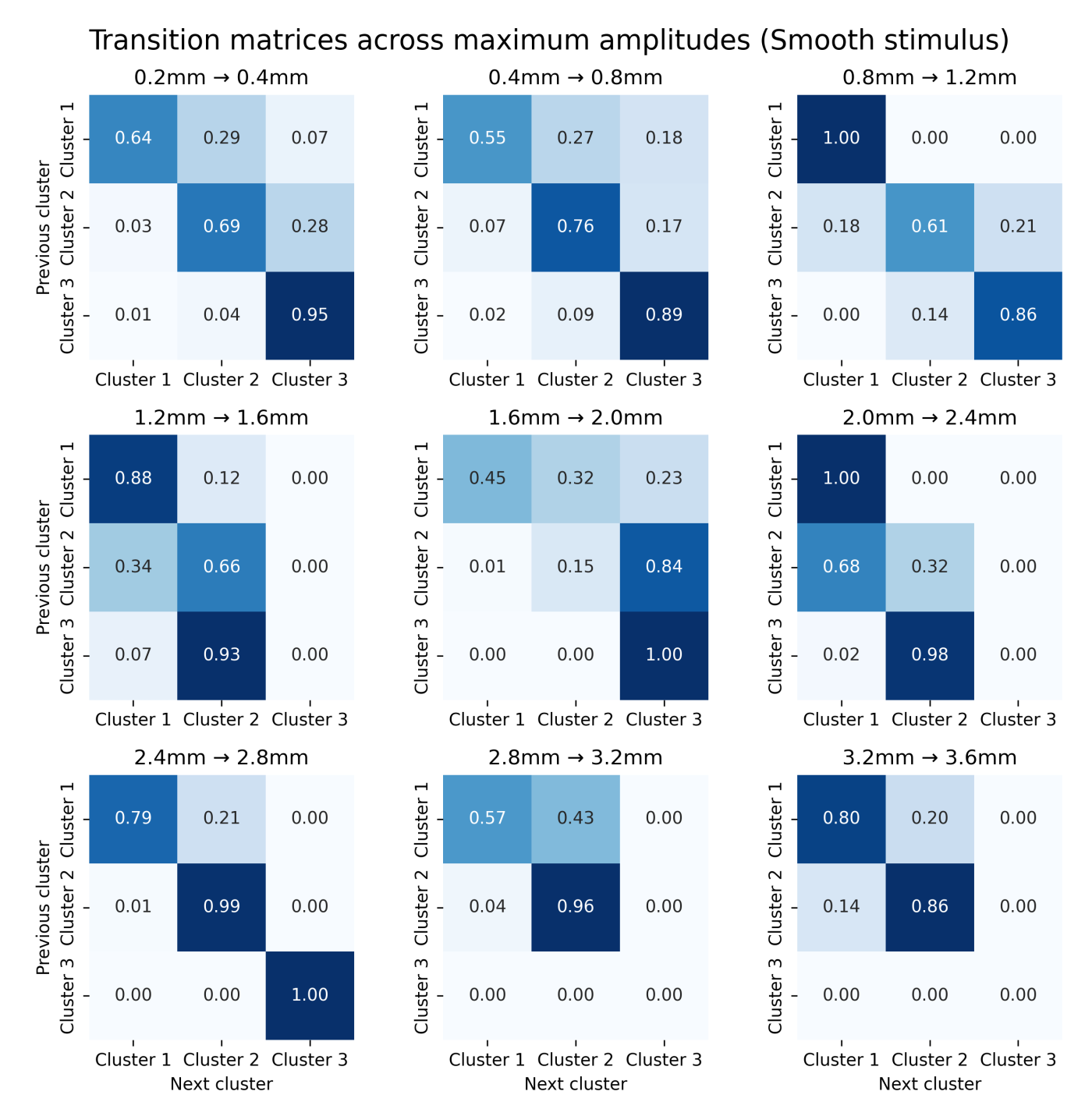

Supplement: S4 Fig — Each heatmap shows the transition probabilities of neurons between clusters across consecutive amplitude levels for the Smooth stimulus. The x-axis represents the cluster assignments at the next amplitude, while the y-axis represents the cluster assignments at the previous amplitude. Colours indicate the probability of neurons transitioning between clusters, with darker shades representing higher probabilities. Each transition matrix illustrates how neurons in Cluster 1, Cluster 2, and Cluster 3 change or maintain their cluster membership as the stimulus amplitude increases from one level to the next (0.2 mm → 0.4 mm, 0.4 mm → 0.8 mm, etc.). (TIF) [file pone.0315887.s004.tif]
