# Supplementary material for: Temporal response patterns of Layer 4 rat barrel cortex neurons across various naturalistic whisker motions
Source: PLoS One. 2024 Dec 23;19(12):e0315887. doi: 10.1371/journal.pone.0315887 (PMC11666059; doi:10.1371/journal.pone.0315887)
Supplement: Attachment — Submitted filename: Response to Reviewers.pdf [file pone.0315887.s005.pdf]

Response to Reviewer for Manuscript Paper PONE-D-24-22742

We would like to thank the editor and reviewers for their constructive comments and suggestions that have helped improve the quality of this manuscript. The manuscript has undergone a thorough revision according to the comments. Please see our responses below. For the reviewers' convenience, we have highlighted significant changes in the revised manuscript in blue and any deletions in red.

Summary of Changes

Below is a summary of the key changes made to the manuscript in response to the reviewer comments:

- We acknowledge the reviewer's suggestion to examine the coherence of cluster membership between the "Rough" and "Smooth" stimuli. However, each experiment was recorded independently, meaning that the neurons tested under each stimulus were not necessarily the same across recordings. As a result, it is not feasible to analyse cluster membership consistency between these stimuli. This clarification has been added to the manuscript.
- A consistency analysis has been added for the "Contact", "Rough", and "Smooth" stimuli, along with transition matrices showing the probability of neurons transitioning between clusters as amplitudes increases. These results support the hypothesis of cluster merging at higher amplitudes and are included in the Results (Figure 6) and Supporting Information section (S2 - S4 Figures).
- We did not separate putative interneurons from excitatory cells due to limitations imposed by the filtering applied during the spike-sorting process. This preprocessing step can obscure the waveform features that would typically distinguish between interneurons and excitatory cells (viz., fast and slow spike waveforms), making reliable classification difficult. This is now discussed in the Methods section when we define our neuronal recordings ("Neurophysiology").
- In response to the variability observed in the 95% confidence intervals and raster plots for the Whisking stimulus, we performed additional clustering analyses at specific time points (150 ms, 175 ms, and 225 ms) to explore whether delayed neuronal responses might reveal distinct clusters. However, the results indicated that only one dominant cluster was present at each time point. These findings suggest that the delayed peaks did not introduce enough variability to drive the formation of new clusters. Confounding factors such as the relatively simple structure of the Whisking stimulus and temporal averaging over the longer duration have been discussed in the manuscript.
- We agree with the reviewer that multiple clusters may emerge when periods of high velocity are temporally compact, as seen with more complex stimuli. Although we did not apply the same stimulus at different time scales, this limitation has been noted, and the Discussion section has been expanded to address the potential effects of temporal compactness and the involvement of different circuits or intrinsic cellular properties in clustering outcomes. However, we have also attempted to address this directly using our dataset, by conducting clustering analyses over different periods of a long stimulus (the Whisking stimulus) ranging

from the full stimulus duration (430 ms) to early shorter periods (150 ms, 175 ms and 225 ms) as detailed in the Methods section ("Single unit spiking activity"). The outcomes of these analyses are reported in the Supporting Information section as S1 Figure and show that the classification, at least for this stimuli, was unaffected by analysis over shorter durations.

- We have made several minor revisions throughout the manuscript to improve clarity, correct figure and panel references, and enhance readability. These changes include addressing specific reviewer feedback related to figure panel numbering, correcting terminology, removing unnecessary figures such as Figure 3 and 5, and adding in S1 - S4 Figures in the Supporting Information section to provide supplementary details.

Major comments

Comment 1 — While I find the study interesting, it would be greatly improved if information about the coherence of clusters between stimuli were provided, where it's possible. Indeed, the numbers of rats and cells reported in Table 1 suggest that "rough" and "smooth" were tested on the same neurons. If this is correct, and because the temporal profiles obtained are similar and the number of clusters is identical, with the same dependence on amplitude, it would be informative to know whether a neuron was in, say, cluster 1 for both "rough" and "smooth". This analysis would also strengthen the GMM clustering that has the lowest Adjusted Rand Index for these stimuli (table 2).

Reply: We appreciate the reviewer's suggestion to examine the coherence of cluster membership across the "Rough" and "Smooth" stimuli. However, each of the experiments in our study was recorded independently, and the neurons tested under each stimulus were not necessarily the same across recordings. This independence between recordings makes it difficult to reliably track the consistency of cluster membership between different stimuli.

As a result, it is not feasible to analyse whether specific neurons remained in the same cluster for both the "Rough" and "Smooth" stimuli. While we acknowledge the value of such an analysis, the experimental setup does not allow for this type of comparison. We have added this limitation to the revised manuscript at the end of the Discussion section.

Comment 2 — Similarly, since different amplitudes were used, for "contact", "rough" and "smooth", it would also improve the study to provide such "consistency index" indicating the extent to which neurons were classified in the same cluster, across amplitudes. In the case of rough and smooth, it would also address the authors' hypothesis that clusters 3 and 2 merge when stimuli of large amplitudes are used (line 566).

Reply: We thank the reviewer for the insightful suggestion regarding the analysis of cluster consistency across amplitudes. In response, we have included a new visualisation (Figure 6), which illustrates the neuron membership across varying amplitudes for the "Contact", "Rough" and "Smooth" stimuli. This figure provides a representation of how individual neurons maintain or shift clusters as stimulus amplitude increases.

Additionally, a transition matrix has been calculated to quantify the probability of neurons transitioning between clusters across different amplitudes, providing a more comprehensive view of how clusters evolve between stimulus amplitudes (S2 - S4 Figure). These additional figures support our hypothesis of

clusters 2 and 3 merging at higher amplitudes for the "Rough" and "Smooth" stimuli. These analyses, along with the new figures, have been incorporated into the revised manuscript.

Comment 3 — Also, separation of putative interneurons from excitatory cells would be a very good addition (give proportions for each cluster?).

Reply: We appreciate the reviewer's suggestion to separate putative interneurons from excitatory cells and provide proportions for each cluster. While such an analysis could offer valuable insights, we encountered challenges in reliably distinguishing between these two cell types.

A key limitation stems from the preprocessing applied after electrophysiological recordings, particularly high-pass filtering, which can alter waveform characteristics essential for neuron classification. This filtering reduces the accuracy of identifying cell types based solely on waveform features, making classification unreliable. Given these limitations, we chose not to pursue this separation in the current study. This clarification has been added to the Method section when we define our neuronal recordings ("Neurophysiology").

Comment 4 — Finally, the fact that GMM did not reveal different populations of neurons for the "basic" and "whisking" stimuli is intriguing. For example, it is difficult to understand why the phasic and prolonged responses are not apparent. What might be the confounding factors? This should be discussed because the patterns obtained with "whisking" do indeed show some variability. This is visible in the 95% confidence interval, which is quite large between peaks, and in the raster plot (Fig 2c, Fig. 5d). In particular, the responses of neurons 1 to 20 seem different from the rest. Besides, for neurons 40-70, one can see some delays in the peaks of activity that are maintained throughout the two cycles of the "whisking" stimulus (times 0.150, 0.175, 0.225 sec...). Could it affect the ability to find clusters?

Reply: We appreciate the reviewer's insightful comment regarding the variability observed in the 95% confidence intervals and raster plots for the Whisking stimulus, particularly the suggestion that the delayed peaks in neuronal responses (at 150 ms, 175 ms and 225 ms) may affect the clustering results.

To address this, we performed additional clustering analyses focused on these specific time points (150 ms, 175 ms and 225 ms) to determine whether the delayed neuronal responses might reveal additional clusters. However, in each case, only a single cluster emerged, suggesting that the delayed peaks in activity did not introduce sufficient variability to generate distinct neuronal populations at these time points (S1 Figure).

Additionally, as discussed in the manuscript, we have outlined potential confounding factors that could influence the ability to detect multiple clusters. These include the relatively simple and uniform structure of the Whisking stimulus, which may not engage diverse sensory processing mechanisms, and the longer duration of the stimulus (430 ms), which could contribute to temporal averaging, thereby masking subtle differences in neuronal responses. The observed delays in certain neurons may introduce temporal variability; however, this variability does not appear to be sufficient to drive the formation of distinct clusters within the time windows analysed.

Comment 5 — Related to the question above, is it possible that multiple clusters can only be found when periods of high velocities are close from each other in the stimulus? Indeed, we see that stimuli that are complex, that produce multiple clusters, are stimuli that are temporally compact (Table 1). It is unfortunate that the same stimulus was not applied at two different time scales (25

ms and 340 ms for example). This would have added to the question of whether the differences in the number of clusters produced by the different stimuli are due to differences in the circuits that are recruited, as proposed in the discussion (lines 697-701), or due to the intrinsic electric properties of the cells, which could also be mentioned in the discussion.

Reply: We appreciate the reviewer's insightful question regarding the possibility that multiple clusters could emerge when periods of high velocities are close together in the stimulus. To address this, we acknowledge that stimuli producing multiple clusters in our data tend to be more temporally compact, which may contribute to distinct neural responses.

We agree that applying the same stimulus at different time scales (e.g., 25 ms and 340 ms) would have been informative in further probing the recruitment of different circuits versus the intrinsic electrical properties of the cells. This limitation has been noted, and we have expanded the end of the Discussion section to address this point.

Minor comments

Comment 6 — Line 55: we have identified that the formation of temporal response patterns is mainly dependent on stimulus complexity and the strength and speed of the whisker deflection. I understand the statement about complexity and strength, but not speed: changing the velocity of the basic stimulus did not allow patterns of responses to be separated.

Reply: We agree with the reviewer and have revised this sentence to clarify the role of speed in stimulus processing. The revised sentence now reads: "We have identified that the formation of temporal response patterns is mainly dependent on stimulus complexity and strength of the whisking deflection."

Comment 7 — Line 71: A sequence of PW deflections was conducted using each of the five stimulus waveforms depicted in Fig. 1. This sentence suggests that multiple stimuli were tested in each recording. Please, correct if it is not the case.

Reply: We have corrected this sentence for clarity. The revised text now states: "A sequence of principal whisker (PW) deflections was conducted using a single stimulus waveform per recording, as depicted in Fig 1." This adjustment clarifies that each stimulus was tested independently.

Comment 8 — Line 82-85: Please, indicate the grain corresponding to rough and smooth.

Reply: We have now included the grain size corresponding to the "Rough" and "Smooth" textures in the Methods section ("Neurophysiology") when we describe each stimuli. The "Rough" texture corresponds to the PW being swept against an 8-grit sandpaper surface, while the "Smooth" texture corresponds to being moved over a glass surface.

Comment 9 — Line 141: Change panel a to b.

Reply: We have corrected the figure reference as suggested. The revised manuscript now refers to the correct panel, changing "panel a" to "panel b".

Comment 10 — Line 149-150: Panels should go from a to e, not a to d. Panel a is for "basic".

Reply: We have corrected the numbering of the figure panels. Panels now run from a to e, and panel a has been correctly associated with the "Basic" stimulus.

Comment 11 — Figure 3: I do not think this figure is necessary.

Reply: We appreciate the reviewer's feedback. We have removed this figure from the manuscript.

Comment 12 — Line 245: Please, describe the method or refer to a paper describing the Dirichlet Process.

Reply: Thank you for the suggestion to include a description of the Dirichlet Process in the manuscript. The revised text outlines how the Dirichlet Process evaluates the statistical similarity of neuronal response patterns. Additionally, we have also provided a mathematical representation of the clustering process to enhance clarity.

Comment 13 — Line 290-297: Overall, the high ARI for the neural responses to these three stimuli suggests that the clustering solutions remained highly consistent, reproducible, and relatively robust across variations in the initialisation seed of the model. The robustness in the optimal number of neural response clusters and in the cluster membership across different clustering solutions provides strong confidence that the identified clusters reliably represent the underlying neuronal temporal response patterns. I would tone down that sentence a bit given that the adjusted Rand Index was high for only 1 of the 3 stimuli that produced clusters.

Reply: We agree that the statement should be toned down. The revised text now reads: "Overall, the relatively high ARI for the Contact stimulus suggests that the clustering solutions remained consistent, reproducible, and relatively robust across variations in the initialisation seed of the model. While the robustness of the clustering model was evident for this stimulus, the optimal number of neural response clusters and the consistency of cluster membership across different solutions provide confidence in the identified clusters. However, further analysis is required to fully assess the stability of the clustering solutions across the Rough and Smooth stimuli."

Comment 14 — Line 373: Correspondingly, the excitatory and inhibitory effects of PC 2 were predominately reflected in Cluster 1. Here, the fast onset excitatory and offset inhibitory activity around 110 ms contributed to the fast onset response and offset responses observed in Cluster 1. It is unclear why we do not see more of the inhibitory activity in the averaged PSTH of cluster 1 neurons. In fact, Cluster 1 neurons appear to be more active than other neurons. They are later described as "Tonic" neurons. Isn't there a contradiction here?

Reply: We appreciate the reviewer's observation regarding the description of inhibitory activity in Cluster 1 and its subsequent classification as a "Tonic" cluster. To clarify, while Cluster 1 does exhibit fast onset excitatory and offset inhibitory activity, the description of "Tonic" refers to the sustained activity throughout the stimulus duration rather than its inhibition at specific moments (such as the offset).

The averaged PSTH of Cluster 1 neurons reflects this sustained activity, which accounts for their higher overall activity compared to other clusters. The inhibitory effect seen around 110 ms primarily influences the offset response, without significantly dampening the sustained activity during the stimulus. This dual behaviour—transient offset inhibition alongside sustained activity—aligns with the

characterisation of Cluster 1 as both exhibiting inhibitory effects and functioning as a tonic response cluster.

We have added this clarification to the revised manuscript to reconcile these observations and to address the perceived contradiction in the interpretation of Cluster 1's activity.

Comment 15 — Figure 5 and lines 388-417: Are these necessary since it was said earlier that GMM yielded a single cluster for "basic" and "whisking" stimuli? The only new information in Fig. 5, namely the 95% confidence interval, could already be shown in Fig. 2, for all stimuli.

Reply: We thank the reviewer's suggestion to consider consolidating the information in Figures 5 and 2. We agree that the 95% confidence interval could be effectively integrated into Figure 2 for all stimuli, thereby reducing redundancy. We have removed Figure 5 and updated Figure 2 with the confidence intervals. We have also updated the descriptions in the Methods section to reflect this change.

Comment 16 — Line 416: Overall, the single temporal response pattern observed in both the Basic and Whisking stimuli demonstrates a high degree of uniformity and synchronicity across all single units, thereby highlighting the robustness of the neuronal responses to these stimuli. There is heterogeneity in the responses to whisking, as visible in the 95% confidence interval and in the raster plot of Fig. 5d and Fig. 2c. In particular the responses from neurons 1 to 20 appear to be different from the rest. Also, one can see some delays in the peaks of activity between neurons 40-70 that are maintained throughout the whisking stimulus (times 0.150, 0.175, 0.225 sec).

Reply: We appreciate the reviewer's insightful comment regarding the heterogeneity observed in the responses to the Whisking stimulus, particularly in neurons 1–20 and 40–70, as reflected in the 95% confidence interval and raster plots (Fig 5d and Fig 2c).

To address this, we have updated the manuscript to acknowledge these variations, particularly those visible in the 95% confidence intervals and the raster plot. In addition, we have closely examined the variability in neuronal responses, particularly at 150 ms, 175 ms and 225 ms, where we observed slight delays in the peaks of activity for some neurons. In response, we performed additional clustering analyses focusing on these specific time points using shortened stimulus durations of 150 ms, 175 ms, and 225 ms to determine if these differences would reveal distinct response patterns. However, no additional clusters were identified, indicating that the overall temporal response pattern remains robust and uniform.

We have revised the manuscript to clarify this analysis and explain how the observed variability was accounted for.

Comment 17 — Line 701: Our observations suggest that this phenomenon is not confined to the cortex and could be present at lower levels of the whisker pathway. What observations?

Reply: We appreciate the reviewer's request for clarification. To address this, we have revised the manuscript by removing the statement "Our observations suggest that this phenomenon is not confined to the cortex and could be present at lower levels of the whisker pathway". Instead, we explain that the reduced diversity observed in Layer 4 responses to simpler stimuli is closely aligned with the preserved encoding of velocity along the whisker pathway.
